# New Technique for Custom-Made Spacers in Septic Two-Stage Revision of Total Hip Arthroplasties

**DOI:** 10.3390/antibiotics10091073

**Published:** 2021-09-04

**Authors:** Moritz Mederake, Ulf Krister Hofmann, Bernd Fink

**Affiliations:** 1 Department of Orthopaedic Surgery, University Hospital Tübingen, Hoppe Seyler-Str. 3, 72076 Tübingen, Germany; ulf.hofmann@med.uni-tuebingen.de; 2 Department of Arthroplasty and Revision Arthroplasty, Orthopaedic Clinic Markgröningen GmbH, Kurt-Lindemann-Weg 10, 71706 Markgröningen, Germany; bernd.fink@rkh-kliniken.de; 3 Orthopaedic Department, University-Hospital Hamburg-Eppendorf, Martinistrasse 52, 20251 Hamburg, Germany

**Keywords:** spacer-periprosthetic joint infection-hip arthroplasty-two-stage revision-antibiotic therapy-orthopedic infections-bone and joint infections

## Abstract

The choice of spacer in the interim phase of two-stage revision hip arthroplasty is crucial. Conventional concepts like a Girdlestone situation, handformed or preformed bone cement spacers show complications like soft-tissue contractions, abrasion of bone cement particles, dislocation, breakage and a low level of mobility in the interim phase. To address these disadvantages, the senior author developed a new technique for custom-made spacers in septic two-stage revision of total hip arthroplasties using prosthetic implants with individualized antibiotic mixture in the cement applying a mechanical inferior cementation method. The aim of this study was to evaluate the results of these spacers with respect to their non-inferiority in terms of reinfection and survival-rate of the new implant and to describe the complications associated with this procedure. Our collective consisted of 130 patients with a median follow-up of nearly five years. With a reinfect-free rate of 92% and a spacer-related complication rate of 10% (8% articular dislocation, 1% periprosthetic joint fracture, 1% breakage), this procedure seems to be safe and superior regarding complications compared to conventional concepts. Further studies are necessary to show the clinical benefit of this procedure.

## 1. Introduction

Periprosthetic infections are serious complications of total hip arthroplasty with an incidence of 1–2% [1,2,3]. In case of a late infection (later than 4 weeks after surgery), all foreign material has to be removed. In such cases, a distinction can be made between one- and two-stage septic revisions. In one-stage septic revision, after removal of all foreign material and radical debridement, a new, usually cemented, prosthesis is implanted in the same operation using antibiotic-containing cement. However, knowledge of the microorganism causing the infection and its antibiogram is essential for this procedure [1,4,5,6]. In selected cases, this concept leads to success rates that are as high as those achieved in two-stage septic revisions [7,8].

The two-stage septic revision includes an initial operation with removal of all foreign material as well as radical debridement and mostly implantation of a spacer loaded with antibiotics. This is followed by an intermediate phase of usually 6 to 12 weeks either with a spacer or with a sine–sine Girdlestone-situation flanked by an antibiotic therapy according to the antibiotic susceptibility profile of the microorganisms detected. Thereafter, an implantation of a prosthesis, either cemented or cementless, is performed, followed by 6 to 12 weeks of the same protocol of antibiotic therapy as in the intermediate phase [1]. The two-stage septic revision is still the most commonly used method for the treatment of periprosthetic late infections. The disadvantage of the two-stage concept is that two surgeries are necessary. The advantage is that surgical debridement is performed twice, with the second operation allowing the eradication of any residual organisms remaining after the first debridement. Antibiotics mostly tailored to the sensitivity of the pathogen are added to the cement of the spacer. Studies between 1994 and 2009 showed success rates of 90% to 100% for two-stage revision concepts for infected hip endoprostheses [9,10,11,12].

The spacer has several functions. The main function of the spacer is to locally release the antibiotic into the infected bed of the prosthesis. Depending on its shape and design, it can also help to minimize soft tissue contractions [9], which can make reimplantation of the new prosthesis in the second step technically easier compared to the Girdlestone situation in which the leg is shortened and where marked formation of scar tissue in the former joint has occurred [1].

There are several different distinctions of spacers: static spacers, which have no articulating surface, or mobile spacers, which form an articulating connection between two spacer parts or the spacer and the debrided bone. Spacers can be preformed (e.g., Spacer G, Tecres, Verona, Italy) or individually manufactured in the operating room (e.g., StageOne Select, ZimmerBiomet, Warsaw, IN, USA or Prostalac, DePuySynthes, Warsaw, IN, USA). All of these concepts have their advantages and disadvantages. 

Mobile spacers can be divided into hemi- and articulating spacers. The hemispacers (only on the femoral side) can be designed as monoblock (e.g., Spacer G, Tecres, Verona, Italy) or modular devices (e.g., StageOne Select, ZimmerBiomet, Warsaw, IN, USA). The disadvantages of these spacers include fracture of the spacer, dislocations and bone resorption at the acetabulum [13,14]. The hemispacer induces bone resorption at the acetabulum because the hard cement has to articulate against the infection-related osteoporotic bone. This is avoided with two-piece articulating spacers by giving the spacer a joint surface of its own. However, this cement-based articular surface in the two-piece spacer can lead to the release of abrasion-induced cement particles, which must be removed during the reimplantation by debridement and synovectomy [15,16].

To address these disadvantages of the described spacer-techniques, a new technique for custom-made spacers in septic two-stage revision of total hip arthroplasties using prosthetic implants with individualized antibiotic mixture in the cement and mechanically inferior cementation as spacers was developed [1,11,15,17,18]. While the idea of these temporary prosthetic implants is to prevent the complications associated with pure cement spacer implantation, their usage means bringing new avital metal and polyethylene surfaces into the joints that were considered septic prior to surgery. The aim of this study was to evaluate the results of these custom-made spacers with respect to their non-inferiority in terms of reinfection and survival-rate of the new implant and to describe the complications associated with this procedure in a larger collective. 

## 2. Material and Methods

### 2.1. Patients

Inclusion criterion was a septic two-stage revision operation with implantation of the custom-made spacer as previously described [1,11,15,17,18]. Exclusion criterion was a follow-up of less than 24 months post-reimplantation. Between April 2013 and October 2017, 141 patients with late periprosthetic infection of the hip endoprosthesis underwent septic two-stage prosthesis revision surgery in the Orthopaedic Clinic Markgröningen. Eleven cases were excluded because of a follow up of less than 24 months. The patient cohort thus consisted of 130 cases with 52 women and 78 men having a median age of 71 (27–92) years, as well as an average body mass index of 29.4 ± 6.1. Diabetes mellitus was known in 24 patients (18%) and a rheumatoid disease in nine patients (7%). Two patients were classified as ASA 1, 59 patients as ASA 2, 67 patients as ASA 3, and 2 patients as ASA 4. The majority of explanted prostheses were cementless, followed by revision implants (Table 1).

In 92 cases, a primary implant was involved, but there were also 33 patients who had already undergone one septic revision. Five patients had already undergone multiple revision operations.

### 2.2. Microbiological Diagnosis

The periprosthetic infection was diagnosed prior to the explantation of the prosthesis in all cases according to the criteria of the Musculoskeletal Infection Society (MSIS) 2014 and ICM 2018 [17,18]. Preoperative aspiration and/or biopsy with microbiological and histological examination of the hip joint was performed; this is a standard procedure in our clinic before any revision of a hip prosthesis is carried out and bacteriological cultivation is assessed for 14 days according to Schäfer et al. [19]. Bacteriological and histological examination according to the methods of Atkins et al. [20], Virolainen et al. [21] and Pandey et al. [22] of the membrane at the site of loosening, which was removed during the operation, was carried out to confirm the original diagnosis.

### 2.3. Surgical Procedure

**Figure 1:**
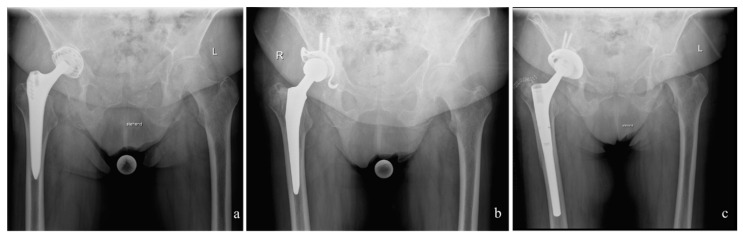
Case with an endofemoral explantation followed by the interim prosthesis and reimplantation. (**a**): Preoperative situation with infected Zweimueller stem. (**b**): After endofemoral explantation of the infected prosthesis, the interim prosthesis was implanted consisting of a cemented stem and acetabular a Ganz-ring with a Müller flat-profile cup (ZimmerBiomet, Winterthur, Switzerland). (**c**): Situation after reimplantation of the definitive prosthesis consisting of Allofit S-cup and Revitan stem (ZimmerBiomet, Winterthur, Switzerland).

After explantation of the infected prosthesis (62 cases endofemoral and 68 cases transfemoral), a radical debridement followed. Thereafter, the custom-made interim prosthesis was implanted. The stem spacer component consisted of a cemented prosthesis stem that was encased in antibiotic-supplemented cement and, just before implantation, was coated in the patient’s own blood in order to facilitate easier removal. The implantation of the cemented spacer was performed with 6 min old cement to reduce to quality of interdigitation of cement to make the removal of the spacer-cement easier in the following operation. The two components of the spacer (femur and acetabulum) were articulated with a metal head (Figure 1).

The acetabular spacer was formed out of a polyethylene cup cemented in either a Müller cup (6 cases), Ganz ring (115 cases), or a Burch-Schneider-acetabular reinforcement ring (9 cases) (ZimmerBiomet, Winterthur, Switzerland), which were fixed with two to maximal four screws and with an individual mixture of antibiotics in the cement according to the susceptibility of the microorganism. 

**Figure 2:**
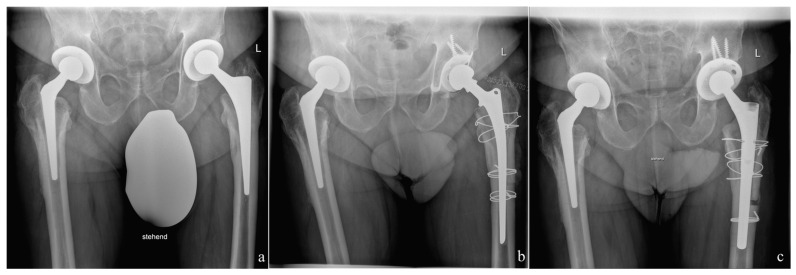
Case with a transfemoral explantation because of a fully osteointegrated prosthetic stem, which was not possible to explant endofemorally. (**a**): Preoperative situation with infected CLS-stem (ZimmerBiomet, Winterthur, Switzerland). (**b**): After transfemoral explantation of the infected prosthesis the interim prosthesis was implanted consisting of a Weber-Stem and acetabular a Ganz-ring with a Müller flat-profile cup (ZimmerBiomet, Winterthur, Switzerland). The Ganz-ring was fixed with only three screws. The transfemoral flap was refixed with 1.5 mm diameter cerclages. (**c**): Situation after reimplantation of the definitive prosthesis consisting of an Allofit S-cup and a Revitan stem (ZimmerBiomet, Winterthur, Switzerland).

In 68 cases, the femoral component was removed via a transfemoral approach when the cementless stem was fully integrated into the bone or the cement mantle was tightly embedded. The transfemoral approach was carried out using a previously published modified Wagner technique [23,24]. Following a posterolateral incision, the posterolateral edge of the femur ventral to the linea aspera was exposed in the septum intermusculare lateral after ligation of the perforating vessels. The lateral circumference of the femur was exposed in the area where the end of the osteotomy flap was going to be positioned and two 3.2 mm holes drilled under cooling (above the linea aspera and 180 degrees ventromedial from the first hole). The ventromedial trochanter region was osteotomized using a chisel at the vasto-gluteal border and then the dorsolateral osteotomy, the connecting osteotomy between the two drill holes and the distal ventromedial osteotomy of about 3 cm were performed with a water-cooled oscillating saw. The ventromedial osteotomy was completed with a chisel that was introduced into the already prepared distal, ventral osteotomy and then driven blind under the vastus lateralis muscle to the proximal end of the osteotomy. The flap with the vastus lateralis muscle attached was opened in a ventromedial direction. If a transfemoral approach had been employed, after the cement-covered stem had been inserted the bony flap was closed immediately with the two double cerclage wires (1.5 mm diameter) and excess cement was removed from the flap (Figure 2). In the second step, the transfemoral approach was opened again and after the implantation of the new prosthetic components, the transfemoral approach was closed again using new double cerclage wires of 1.5 mm diameter.

### 2.4. Applied Bone Cement and Administered Antiinfective Substances

All cases underwent bacteriological examination prior to the revision surgery according to the methods of Atkins et al. [20], Virolainen et al. [21] and Pandey et al. [22]. According to the anti-infective susceptibility profile of the microorganisms, a specific mixture of anti-infective substances was applied to the bone cement according to a microbiologist’s suggestion. To avoid mechanical problems with the bone cement, a maximum of 10% of the total cement powder weight was added as anti-infective substance (e.g., 2 g Vancomycin plus 1 g Clindamycin plus 1 g Gentamycin in 40 g Copal cement). As industrially prepared cement Copal cement [Heraeus, Darmstadt, Germany] was used. Since not all anti-infective substances are suitable to be added to PMMA bone cement, the range of substances to choose from was based on the recommendations of the PRO-IMPLANT-Foundation as described by Kühn et al., 2017 [25]. The cement of the spacer contained two anti-infective substances in 63 cases, three in 62 cases and four in 5 cases (Table 2).

### 2.5. Post-Operative Regime

After spacer implantation, the patients were discharged after two weeks of parenteral antibiotic therapy and mobilization with partial weight bearing on the operated leg. There was no restriction in range of motion for the operated joint beside the avoidance of movements hazardous for dislocation. The parenteral anti-infective therapy was administered specifically for each case according to a microbiologist’s suggestion and initiated during surgery once the implant had been removed, the infected and ischemic tissues had been effectively debrided, and at least 5 samples of tissue had been obtained for the microbiological assessment (14 days of enrichment) from the joint capsule and from the membrane around the loosened region as well as from the purportedly infected tissues. 

Anti-infective treatment was performed for six weeks after the first stage surgery.

During the re-implantation procedure, at least five samples of tissue were removed for bacteriological examination. Antibiotic treatment followed the same protocol as after the first operation. 

After re-implantation of the new prosthesis, the leg was subjected to partial weight bearing by loading with 10 kg for a period of six weeks. Thereafter, the weight bearing was gradually increased to full weight bearing 3 months postoperatively as previous described by other authors for other cementless revision stems [11,25,26,27].

### 2.6. Follow-up

All patients were examined before the operation and then 3 months, 6 months, 9 months, one year, 18 months and two years after the operation and at the latest follow-up. Inflammatory parameters (C-reactive protein) were also followed. According to Diaz-Ledezma et al. [26] a patient could be judged infection-free at follow-up if he or she was free from mortality related to periprosthetic joint infection, free from subsequent surgical intervention for periprosthetic joint infection and if there was a microbiological and clinical absence of the infection for at least 2 years. The suspicion of a periprosthetic joint infection was again ruled out or confirmed with the MSIS criteria 2014 and the ICM criteria 2018. In the event of one of these criteria and a confirmed periprosthetic joint infection, a reinfection was assumed.

### 2.7. Statistical Analyses

Statistical analyses were conducted using IBM SPSS Version 20 (IBM, Armonk, NY, USA) and Microsoft Excel (Microsoft, Redmond, WA, USA). Distributions of variables within the groups were assessed by histograms and a non-parametric approach was chosen. Continuous variables are presented as medians and ranges, and categorical variables as frequencies. Survival is presented with a Kaplan–Meier curve. 

### 2.8. Ethical Approval

The study was conducted according to the guidelines of the Declaration of Helsinki, and was approved by the local ethics board of the Ärztekammer Nordwürttemberg (registration number F-2014-021).

## 3. Results

### 3.1. Microbiological Etiology

Most frequently detected were *Staphylococcus epidermidis*, *Cutibacterium acnes* and *Staphylococcus aureus* (Table 3) with Gram-positive bacteria involved in 72% of all cases. It should be noted that among all infected cases, in three cases there was no causative microorganism detected, in 24 cases (18%) two different causative organisms were identified, and in 1 case even three microorganisms were found positive (Supplementary Data S1). 

### 3.2. Antiinfective Therapy

All patients were administered parenteral anti-infective therapy for 2 weeks, starting perioperatively at the first stage operation. The high bioavailability of the antibiotics rifampicin and ciprofloxacin allowed their oral administration from the second day following surgery. There were 65 cases where one antibiotic or antimycotic substance was administered systemically, 60 cases which received two anti-infective substances and four cases with four anti-infective substances. One patient was administered six anti-infective substances because of a mycobacterial infection (Table 4).

Parenteral antibiotic therapy was followed by orally administered anti-infectives for four weeks, having an anti-infective treatment of 6 weeks before performing the second stage. There was no set time between the end of the first anti-infective therapy and the second stage surgery. Two antibiotics or antimycotics were administered in 73 cases, three antibiotics or antimycotics in 2 cases and five antibiotics in 1 case (Table 5).

Anti-infective treatment followed the same protocol as after the first operation being finished six weeks postoperatively.

### 3.3. Follow-up

Median follow-up until dropout was 51 (24–92) months. Reasons for dropout were reinfection (11 cases), death (7 cases) or end of follow-up (112 cases). No perioperative deaths were observed in the collective and the reported drop-outs due to death were not directly linked to septicemia associated with the infected prothesis.

At the end of the follow-up, 119 cases (92%) could be classified as “free of reinfect” and 11 cases (8%) had to be classified as “reinfected”. Four of the eleven reinfected cases had a prior septic revision surgery (Table 6).

**Figure 3:**
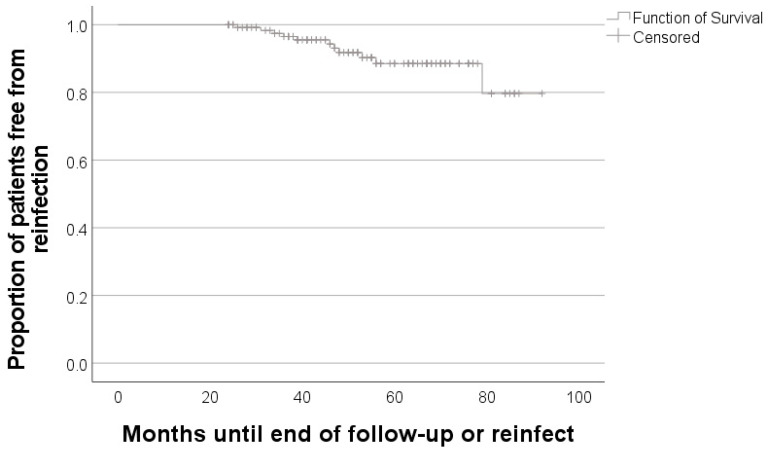
Kaplan–Meier curve for the proportion of patients free from reinfection.

Mean time of survival after the two-stage revision operation was 85 (95%-CI 81–89) months (Figure 3).

In eight cases (6%), the samples taken during the second operation were positive for bacterial infection. *Staphylococcus epidermidis* was detected in three cases (2.5%), *Staphylococcus aureus* in a further three cases (2.5%) and *Staphylococcus capitis* in two cases (1%). However, in each case, there was only just one positive culture out of at least 5 taken samples. This is why these samples were considered as contamination. None of the cases with a positive detection of microorganisms at the time of the second operation had a reinfect during follow-up. 

### 3.4. Complications

Spacer-related complications occurred in 13 cases (10%) with the most frequent complication being articular dislocation in 11 cases (8%). Besides dislocation, there was one periprosthetic fracture (1%) and one cup breaking out (1%). Revision operation of the spacer was necessary in eleven cases (8%).

## 4. Discussion

The implantation of spacers, especially mobile spacers, in the interim phase of two-stage revision makes reimplantation easier and helps to maintain the patient’s mobility [9]. The technique described in the present work was developed in order to combine the advantages of a single-stage procedure of a prosthetic implantation with the radical debridement of the two-stage procedure. Due to the specific anti-infective local and systemic treatment, it was assumed that, despite implanted prosthesis parts (e.g., polyethylene cup) in the interim period, similarly high rates of freedom from infection would be achieved [7,8].

Furthermore, this method benefits from the implantation of stable implants and a tribologically good articulation. One advantage of the method described is the avoidance of cement particle abrasion through tribological implant articulation. This procedure is also suitable for situations after transfemoral explantation, as correspondingly long prosthesis stems can be used. By using different cups (e.g., Ganz ring, Burch–Schneider ring), acetabular defects can also be adequately addressed. In addition, a combined procedure (one-stage cup change and two-stage stem change) is possible with this method [28]. Last but not least, the procedure offers the antimicrobial advantage that an additional and tested antibiotic can be added individually to the cement used.

The present study was carried out to evaluate the success rates and survival time after reimplantation. In addition, spacer-specific complications should be investigated. The aim of the study was to assess whether the present method does not have any disadvantage compared to conventional spacers.

The procedure described herein with implantation of a custom-made interim prosthesis results in a reinfect-free rate of 92% with a mean follow-up time of nearly 5 years. These results are in line with existing literature using other spacer techniques [27,29,30,31]. A standardized procedure with a Girdlestone-situation showed a reinfect-free rate of 89% [27]. Chen et al. were able to reach a reinfect-free rate of 91% with a follow-up of over 9 years. The spacer technique was a hand-made interim antibiotic-impregnated articulating polymethyl methacrylate spacer [29]. Ibrahim et al. evaluated a mixed collective with mobile and static spacers and reached a reinfect-free rate of 96% [30]. In a large-scale retrospective study, Triantafyllopoulos et al. reached with approximately 92%, an almost identical reinfect-free rate as in our collective [31]. The main difference to conventional methods, which could affect the risk of reinfection is the implantation of metal and polyethylene prostheses parts. However, regarding the microbiology at the time of the reimplantation of the prosthesis, only 6% of the samples taken were positive. Of note, all of these cases had only one positive out of at least five taken samples classifying these positive samples as contamination. Compared to studies investigating the value of reimplantation microbiology showing a positive result in 5–14% our results are comparable or even lower. Furthermore, these studies show that the reimplantation microbiology is not suitable to predict the risk of reinfection [32,33]. 

Our results imply that although parts made of metal and polyethylene are implanted in the interim phase, there are no infectiological disadvantages compared to conventional methods. 

With 10%, our complication rate can be classified as very low when comparing with other studies ranging at 26% or even higher [34,35,36]. Having a closer look at the complications, the most frequent complication in our collective was the articular dislocation with 8%. Although the patient is allowed a full range of motion and it is a regular tribological articulation, this rate is lower than in comparable studies with a range of 9–42%. The breaking out of the cup and the periprosthetic femur fracture, each with only one case (1%) in our collective, is also less than with conventional spacers ranging at 20% [35,36]. This fact is certainly due to the cementation of stable implants.

The disadvantage compared to conventional hand-formed spacers made of cement are the higher costs caused by the implants. More precisely, there are costs not only for the cement used, but also for the additional prosthesis parts that are implanted. The exact amount of the higher costs is difficult to estimate, as the prices can vary greatly depending on the negotiations with the manufacturer. Since this is a temporary interim prosthesis, it is possible to fall back on tried and tested prostheses that have been available on the market for a long time in order to save costs. When choosing the prosthesis, the most important aspect seems to be the high-quality tribological articulation. This is also guaranteed with standard models that have been on the market for some time. New models, for example with special surface coatings, modular prostheses or even custom-made products might develop their advantages in long-term use, which is not relevant in the six week period of interim spacers.

One clear strength of the study is the high number of patients evaluated. This offers the opportunity of giving a very reliable statement to the key questions. Furthermore, the consistent treatment regime without having different concepts in comparison gives our results more weight.

A key limitation of our study is the retrospective character of the analysis with its well-known weaknesses. In addition, as described, the clinical advantages of our method can be assumed, but these are not scientifically proven in the present study by clinical scores. Further studies to evaluate the clinical benefit must therefore follow. Such a study is already planned in our clinic.

## 5. Conclusions

In this study, we were able to show that using customized interim protheses with regular implant tribology does not lead to increased infection or complication rates while at the same time allowing a free range of motion in the interim phase between two-staged revision surgery. To ascertain the clinical advantage over conventional spacers, further studies with recording of clinical scores must be carried out. Such a study is already being planned at our center. Although first results are promising, we do not have enough data yet to give a sufficient statement.

## Figures and Tables

**Table 1:** Types of explanted prostheses.

		Number	%
Primary implant	Cementless total hip arthroplasty	60	45
	Hybrid total hip arthroplasty	19	15
	Cemented total hip arthroplasty	7	5
	Bipolar prosthesis	2	2
	Surface replacement prosthesis	2	2
Revision implant		40	31

**Table 2:** Spacer cement and the individual mixture of added anti-infective substances.

Spacer Cement	Individually Added Antiifective Substances	Number
Copal ^1^ G + C (Gentamycin + Clindamycin)	Vancomycin	58
Copal G + V (Gentamycin + Vancomycin)		36
Copal G + C (Gentamycin + Clindamycin)		27
Copal G + C (Gentamycin + Clindamycin)	Vancomycin, Meropenem	4
Copal G + C (Gentamycin + Clindamycin)	Meropenem	3
Copal G + C (Gentamycin + Clindamycin)	Streptomycin	1
Copal G + C (Gentamycin + Clindamycin)	Vancomycin, Amphotericin	1

^1^ Heraeus, Darmstadt, Germany.

**Table 3:** Identified microorganisms and the number of detections.

Classification	Microorganism	Number	% of Cases Infected by This Pathogen
Gram-positive cocci (total in 93 cases/72%)	*Staphylococcus epidermidis*	38	29
	*Staphylococcus aureus*	19	15
	*Staphylococcus capitis*	6	5
	*Staphylococcus lugdunensis*	3	2
	*Staphylococcus hominis*	3	2
	*Staphylococcus warneri*	3	2
	*Staphylococcus caprae*	2	2
	*Staphylococcus haemolyticus*	2	2
	*Staphylococcus saccharolyticus*	2	2
	*Staphylococcus saprophyticus*	1	1
	*Streptococcus salivarius*	3	2
	*Streptococcus agalactiae*	1	1
	*Streptococcus gordonii*	1	1
	*Streptococcus anginosus*	1	1
	*Streptococcus mitis/oralis*	1	1
	*Enterococcus faecalis*	7	5
Gram-positive rods (total in 46 cases/35%)	*Cutibacterium acnes*	33	25
	*Cutibacterium granulosum*	9	7
	*Listeria monocytogenes*	2	2
	*Lactobacillus plantarum*	1	1
	*Actinomyces odontolyticus*	1	1
Gram-negative rods (total in 11 cases/8%)	*Escherichia coli*	5	4
	*Klebsiella pneumoniae*	2	2
	*Enterobacter aerogenes*	1	1
	*Bacteroides fragilis*	1	1
	*Proteus mirabilis*	1	1
	*Morganella morganii*	1	1
Atypical gram behaviour (total in 1 case/1%)	*Mycobacterium tuberculosis*	1	1
Fungal pathogen (total in 2 cases/2%)	*Candida albicans*	2	2

**Table 4:** Intravenously administered anti-infective substances or combination of anti-infective substances with number of patients.

Antibiotic 1	Antibiotic 2	Antibiotic 3	Number
Amoxicillin/Sulbactam			32
Vancomycin	Rifampicin		27
Flucloxacillin			23
Levofloxacin	Rifampicin		5
Vancomycin	Fosfomycin		4
Cefuroxime			3
Meropenem	Ciprofloxacin		3
Vancomycin	Imipenem		3
Flucloxacillin	Piperacillin/Tazobactam		2
Imipenem			2
Penicillin G			2
Penicillin V			2
Amoxicillin	Rifampicin		1
Amoxicillin			1
Ampicillin/Sulbactam	Clindamycin		1
Ampicillin/Sulbactam	Ethambutol, Pyrazinamide, Amicacin, Rifabutin and Moxifloxacin		1
Ampicillin/Sulbactam	Metronidazole		1
Ampicillin/Sulbactam	Vancomycin	Fosfomycin	1
Ceftriaxone			1
Cephazolin	Clindamycin		1
Cotrimoxazole	Rifampicin	Amphotericin B	1
Daptomycin			1
Flucloxacillin	Rifampicin	Amphotericin B	1
Fosfomycin	Imipenem	Vancomycin	1
Fosfomycin	Ampicillin/Sulbactam		1
Fosfomycin	Flucloxacillin		1
Fosfomycin	Meropenem		1
Imipenem	Ciprofloxacin		1
Levofloxacin	Metronidazole		1
Meropenem	Levofloxacin		1
Moxifloxacin	Flucloxacillin		1
Vancomycin	Meropenem		1
Vancomycin	Piperacillin/Tazobactam		1
Voriconazole			1

**Table 5:** Administered oral antibiotics or antimycotics or combination and number.

Antibiotic 1	Antibiotic 2	Antibiotic 3	Number
Levofloxacin	Rifampicin		50
Amoxicillin/clavulanic acid			29
Cotrimoxazole	Rifampicin		8
Linezolid			5
Clindamycin			4
Ciprofloxacin			4
Amoxicillin/clavulanic acid	Levofloxacin		3
Cotrimoxazole			3
Moxifloxacin			2
Ciprofloxacin	Linezolid		2
Clindamycin	Rifampicin		2
Linezolid	Rifampicin		2
Stopped because of elevated liver parameters			3
Ampicillin/Sulbactam			2
Voriconazole			1
Ethambutol, Pyrazinamide, Amicacin, Rifabutin and Moxifloxacin			1
Amoxicillin/clavulanic acid	Metronidazole		1
Moxifloxacin	Rifampicin		1
Stopped because of linezolid allergy (linezolid was only sensitive antibiotic)			1
Cefuroxime	Clindamycin		1
Levofloxacin	Metronidazole		1
Cotrimoxazole	Fluconazole		1
Amoxicillin/clavulanic acid	Rifampicin	Levofloxacin	1
Cotrimoxazole	Rifampicin	Levofloxacin	1
Levofloxacin	Clindamycin		1

**Table 6:** Details of cases with persistent infection or reinfection.

Infected Case Number	Prior Septic Revision Surgery	Causative Microorganism at the Time of Revision Surgery	Causative Microorganism at the Time of Reinfect
1.	No	*Staphylococcus aureus*	Not known
2.	Yes	*Staphylococcus capitis*	*Staphylococcus aureus, Corynebacterium urealyticum*, *Cutibacterium acnes*
3.	Yes	*Cutibacterium acnes*	*Cutibacterium granulosum*
4.	Yes	*Staphylococcus capitis, Cutibacterium acnes*	*Staphylococcus epidermidis*
5.	Yes	*Cutibacterium acnes*	Not known
6.	Yes	*Staphylococcus epidermidis*	*Cutibacterium granulosum*
7.	Yes	*Cutibacterium acnes*	*Staphylococcus capitis*
8.	Yes	*Cutibacterium acnes*	*Staphylococcus aureus*
9.	No	*Enterococcus faecalis*	Not known
10.	No	*Candida albicans*	*Staphylococcus epidermidis*
11.	No	*Escherichia coli*	*Escherichia coli*

## Data Availability

The data presented in this study are available on request from the corresponding author.

## Supplementary Materials

The following are available online at https://www.mdpi.com/article/10.3390/antibiotics10091073/s1, Data S1: Combination of causative microorganisms in polymicrobial infections and number of occurrence.
