# Supplementary material for: New Technique for Custom-Made Spacers in Septic Two-Stage Revision of Total Hip Arthroplasties
Source: Antibiotics (Basel). 2021 Sep 4;10(9):1073. doi: 10.3390/antibiotics10091073 (PMC8469632; doi:10.3390/antibiotics10091073)
Supplement: Supplementary file 1 [file antibiotics-10-01073-s001.zip › antibiotics-1338026-supplementary.pdf]

Supplementary Materials

Data S1: Combination of causative microorganisms in polymicrobial infections and number of occurrence

	**Causative microorganism 1**	**Causative microorganism 2**	**Causative microorganism 3**	**Causative microorganism 4**	**Number**
1.	*Staphylococcus epidermidis*	*Cutibacterium acnes*			3
2.	*Staphylococcus capitis*	*Cutibacterium acnes*			3
3.	*Staphylococcus hominis*	*Cutibacterium acnes*			2
4.	*Staphylococcus epidermidis*	*Cutibacterium granulosum*			2
5.	*Staphylococcus saprophyticus*	*Staphylococcus warneri*			1
6.	*Staphylococcus lugdunensis*	*Cutibacterium acnes*	*Enterococcus faecalis*		1
7.	*Staphylococcus haemolyticus*	*Cutibacterium acnes*			1
8.	*Staphylococcus haemolyticus*	*Enterococcus faecalis*			1
9.	*Staphylococcus epidermidis*	*Staphylococcus lugdunensis*			1
10.	*Staphylococcus epidermidis*	*Staphylococcus aureus*			1
11.	*Staphylococcus epidermidis*	*Staphylococcus hominis*			1
12.	*Staphylococcus epidermidis*	*Staphylococcus caprae*			1
13.	*Staphylococcus epidermidis*	*Enterococcus faecalis*			1
14.	*Staphylococcus capitis*	*Cutibacterium granulosum*			1
15.	*Staphylococcus capitis*	*Staphylococcus saccharolyticus*			1
16.	*Cutibacterium granulosum*	*Mycobacterium tuberculosis*			1
17.	*Enterococcus faecalis*	*Staphylococcus aureus*			1
18.	*Escherichia coli*	*Klebsiella pneumoniae*	*Enterococcus faecalis*	*Morganella morganii*	1
19.	*Escherichia coli*	*Staphylococcus epidermidis*			1
20.	*Staphylococcus warneri*	*Actinomyces odontolyticus*			
